# Supplementary material for: Lipocalin-2, Matrix Metalloproteinase-9, and MMP-9/NGAL Complex in Upper Aerodigestive Tract Carcinomas: A Pilot Study
Source: Cells. 2025 Mar 29;14(7):506. doi: 10.3390/cells14070506 (PMC11988122; doi:10.3390/cells14070506)
Supplement: Supplementary file 1 [file cells-14-00506-s001.zip › cells-3474848-supplementary.pdf]

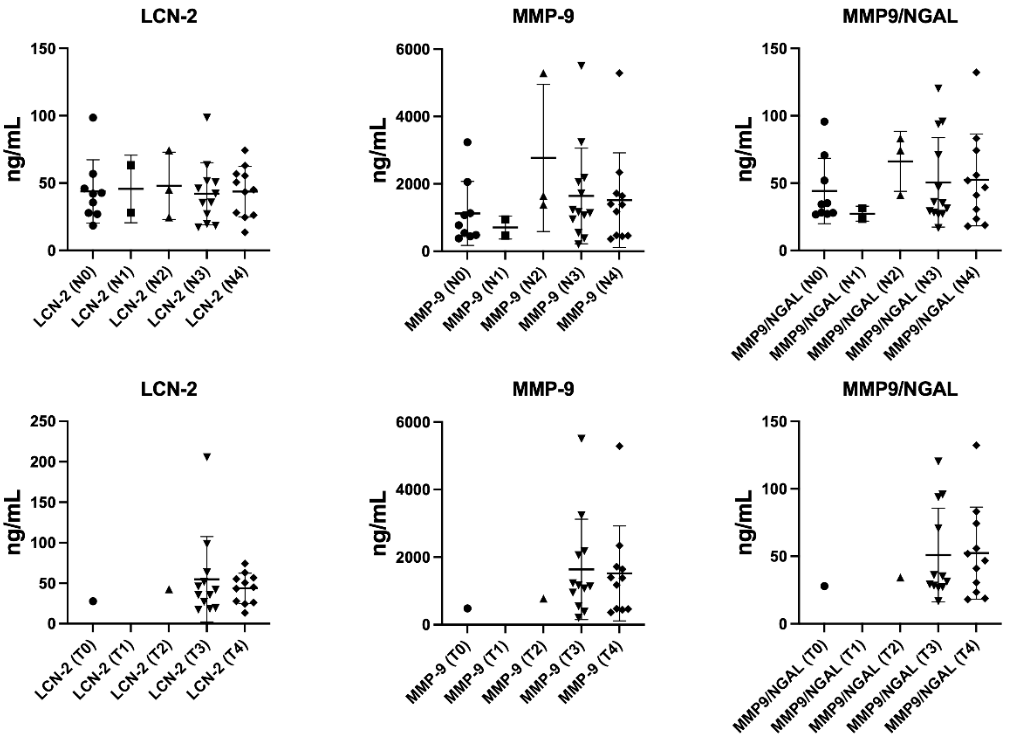

Figure S1. LCN-2, MMP-9 and MMP-9/NGAL complex circulating levels UADT cancer patients, stratified following number of lymph node involved (N0-1-2-3-4) (upper panels) and tumor stage (T0-1-2-3-4). Scatter dot plots represent the mean ± SD of serum levels UADT patients.

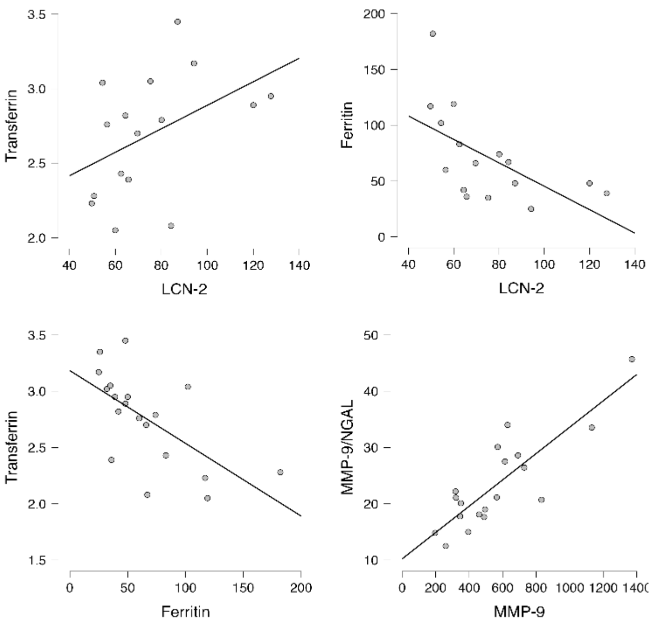

Figure S2. Scatter plot graphs reporting significant Spearman's Correlations in healthy controls (see Table 3 for details on *pho* and p-values).

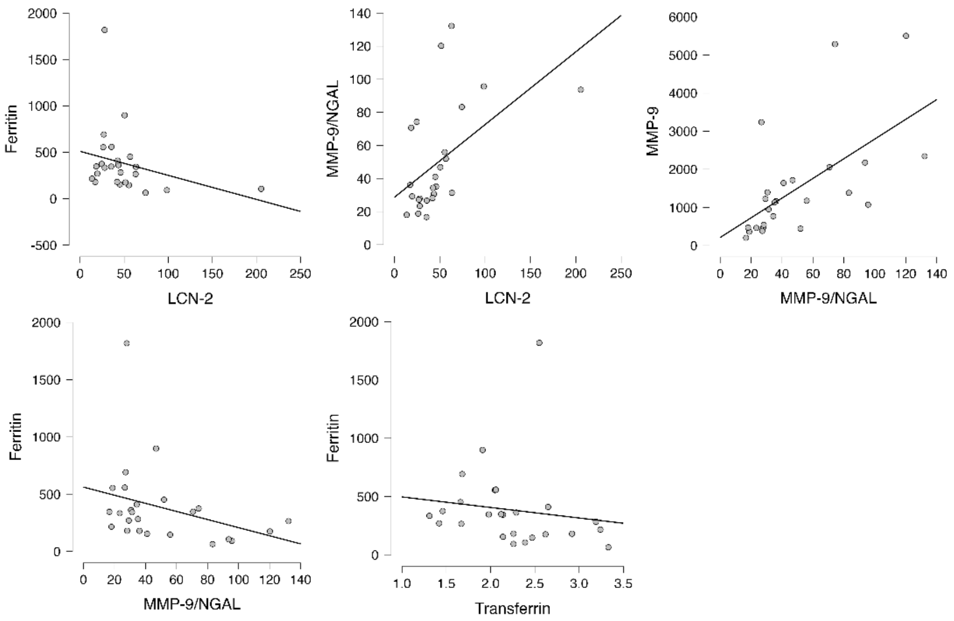

Figure S3. Scatter plot graphs reporting significant Spearman's Correlations in UADT cancer patients (see Table 3 for details on *pho* and p-values).
